# Supplementary material for: Negative magnetoresistance without well-defined chirality in the Weyl semimetal TaP
Source: Nat Commun. 2016 May 17;7:11615. doi: 10.1038/ncomms11615 (PMC4873626; doi:10.1038/ncomms11615)
Supplement: Supplementary Information — Supplementary Figures 1-18, Supplementary Table 1, Supplementary Notes 1-6 and Supplementary References [file ncomms11615-s1.pdf]

Supplementary Figures

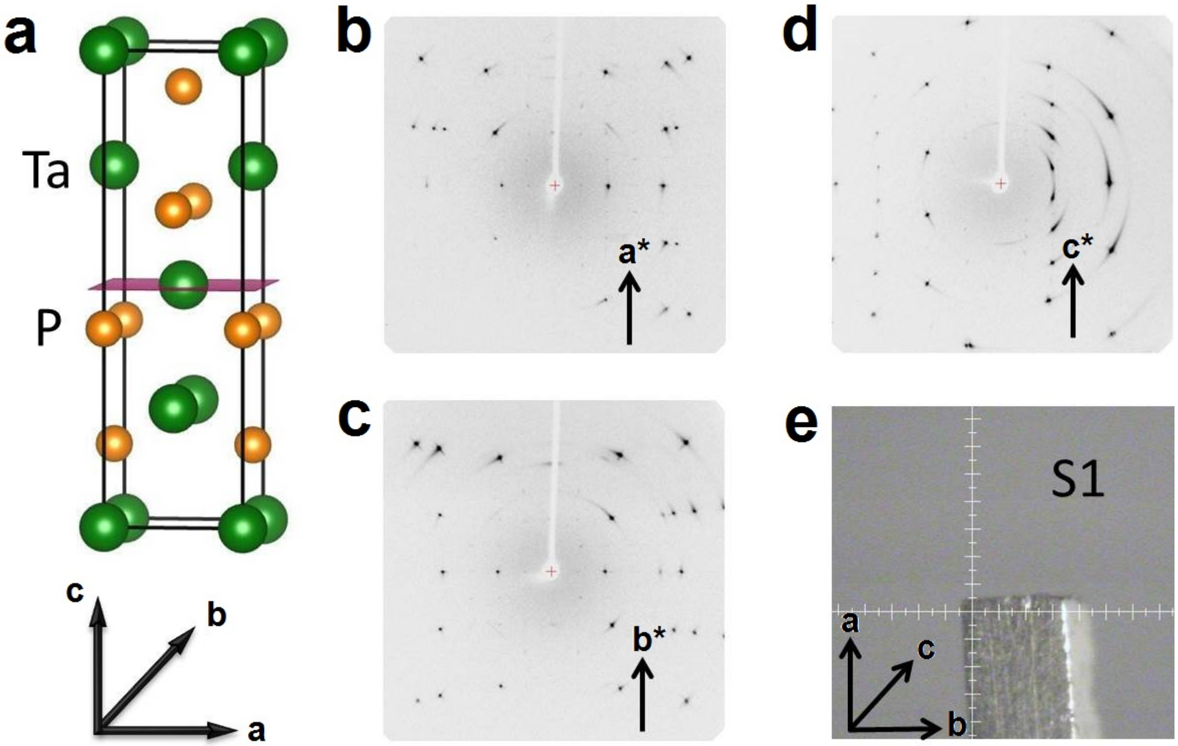

Supplementary Figure 1: Unit cell of TaP and x-ray diffraction patterns of crystal S1. (a), non-centrosymmetric unit cell of TaP. **(b)**, **(c)** and **(d)**, show the rotation diffraction patterns of the TaP crystal, S1, for the rotation about the crystallographic **a**, **b**, and **c**-axis respectively. The rotation axis in each case is vertical. **(e)**, shows an image of S1 with its longest dimension parallel to the crystallographic **a**-axis.

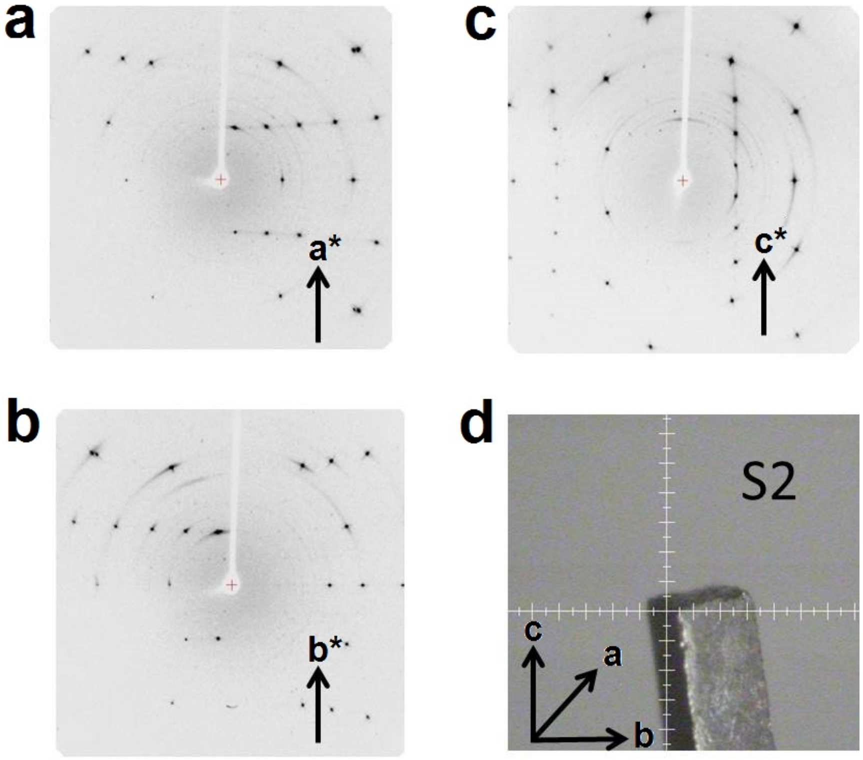

Supplementary Figure 2: **X-ray diffraction patterns of crystal S2.** (**a**), (**b**) and (**c**), show the rotation diffraction patterns of the TaP crystal, S2, for the rotation about the crystallographic **a**, **b**, and **c**-axis respectively. The rotation axis in each case is vertical. **(d)**, shows an image of S2 with its longest dimension parallel to the crystallographic **c**-axis.

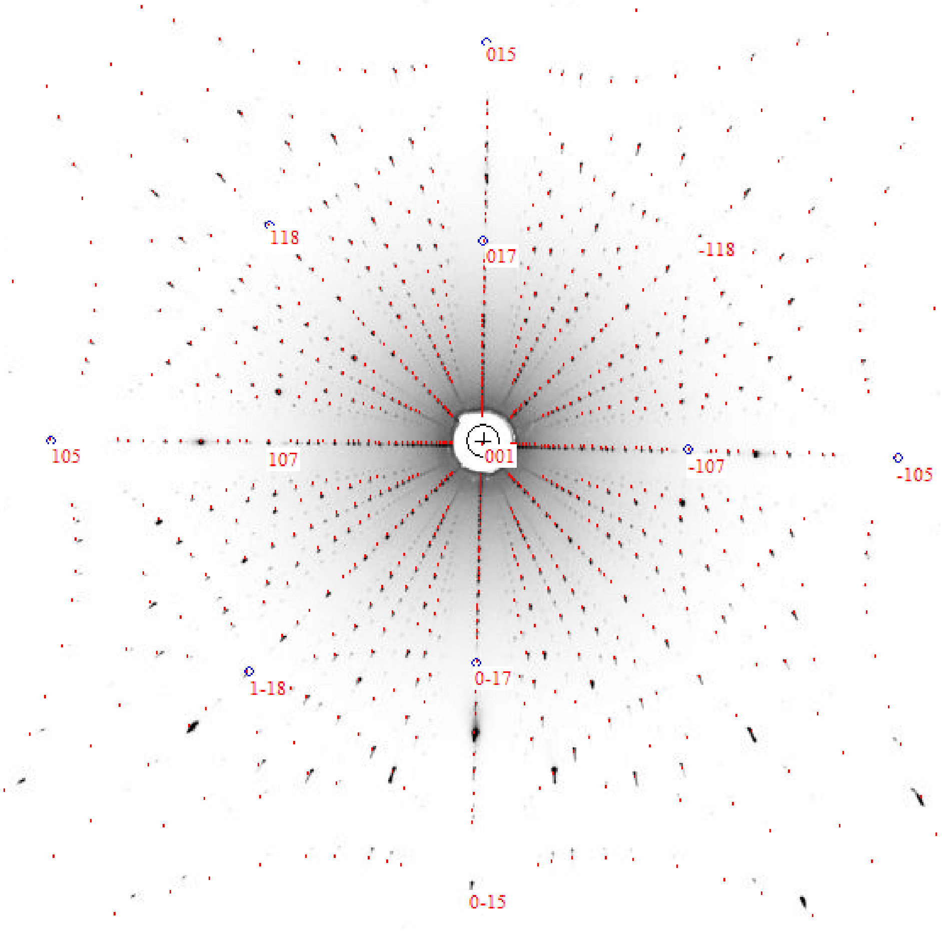

Supplementary Figure 3: **Laue diffraction image of TaP**. The graph shows the room temperature Laue diffraction image of an as-grown (100) facet of a TaP single crystal. The red spots and assigned Miller indices show the calculated diffraction pattern of the $I4_1md$ tetragonal-space group.

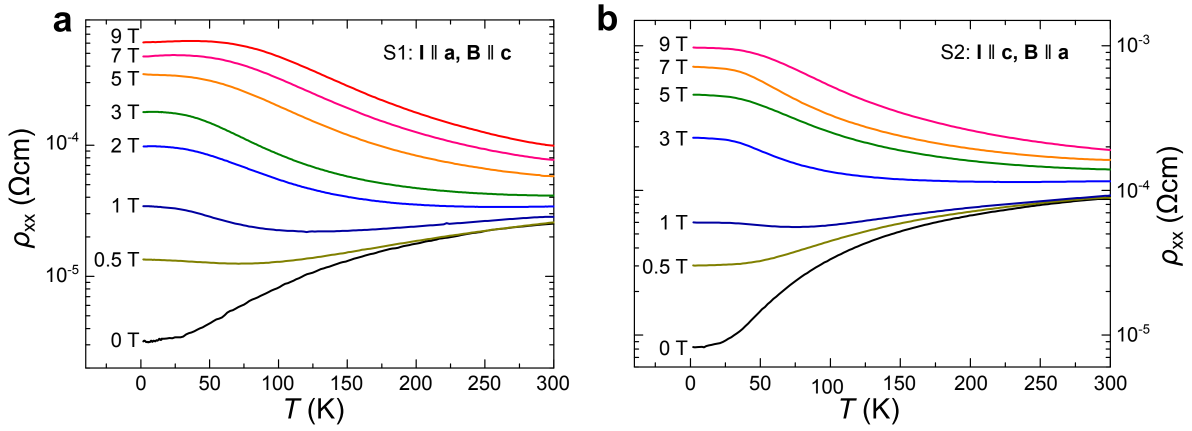

Supplementary Figure 4: Temperature dependence of transverse resistivity. **(a)** shows the transverse resistivity, ρ_{xx}, of sample S1 measured along the **a** axis up to 9 T and **(b)** of sample S2 measured along the **c** axis for **B**⊥**I**. At zero magnetic field, the resistivities decrease monotonically with decreasing temperature. At moderate magnetic fields of about 1 T and intermediate temperatures a crossover from a positive to a negative temperature coefficient takes place.

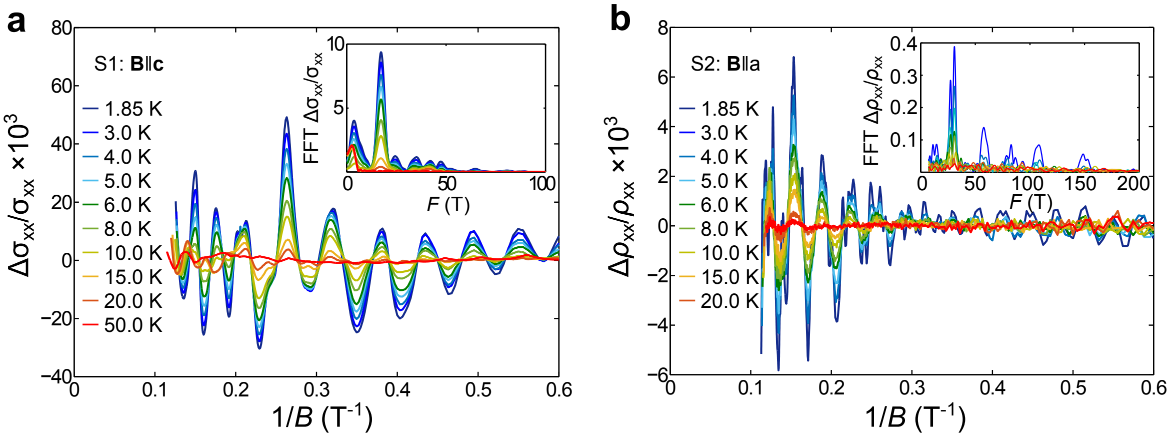

Supplementary Figure 5: Shubnikov-de Haas oscillations at different temperatures **(a)** shows the Shubnikov-de Haas oscillations plotted against $1/B$ for **B**∥**c**, **(b)** for **B**∥**a**. The insets show the temperature dependent Shubnikov-de Haas Fourier transforms.

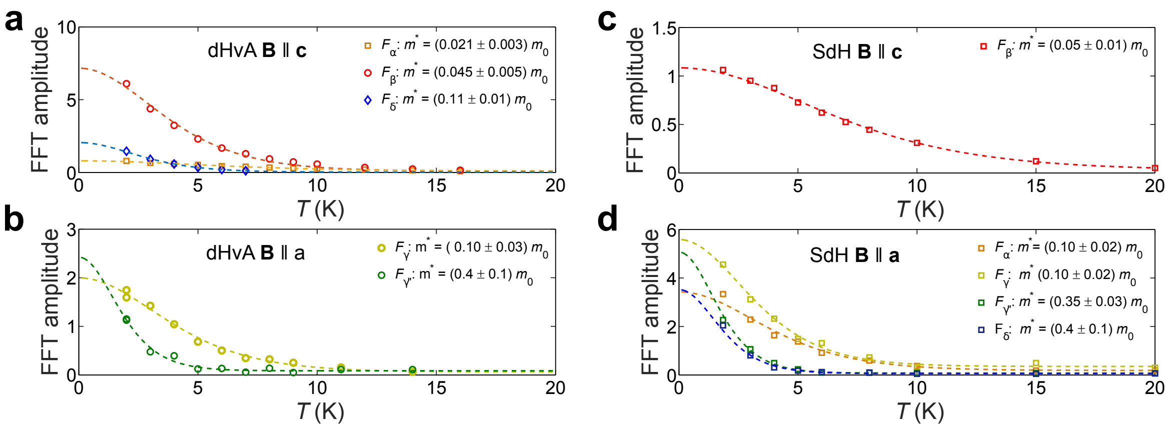

Supplementary Figure 6: Effective masses from de Haas-van Alphen and Shubnikov-de Haas amplitudes. **(a)** and **(b)** show the temperature dependence of the de Haas-van Alphen Fourier transform amplitudes in **B**||**c** and **B**||**a** direction respectively, as determined from SQUID-VSM measurements and the corresponding Lifshitz-Kosevich temperature reduction term fits [1,2]. **(c)** and **(d)** show the Shubnikov-de Haas amplitudes and temperature reduction term fits of samples S2 for **B**||**c** and **B**||**a** direction (**(c)** and **(d)** respectively). In order to increase the resolution of our data, different magnetic field ranges were used to calculate the Fourier transform amplitudes.

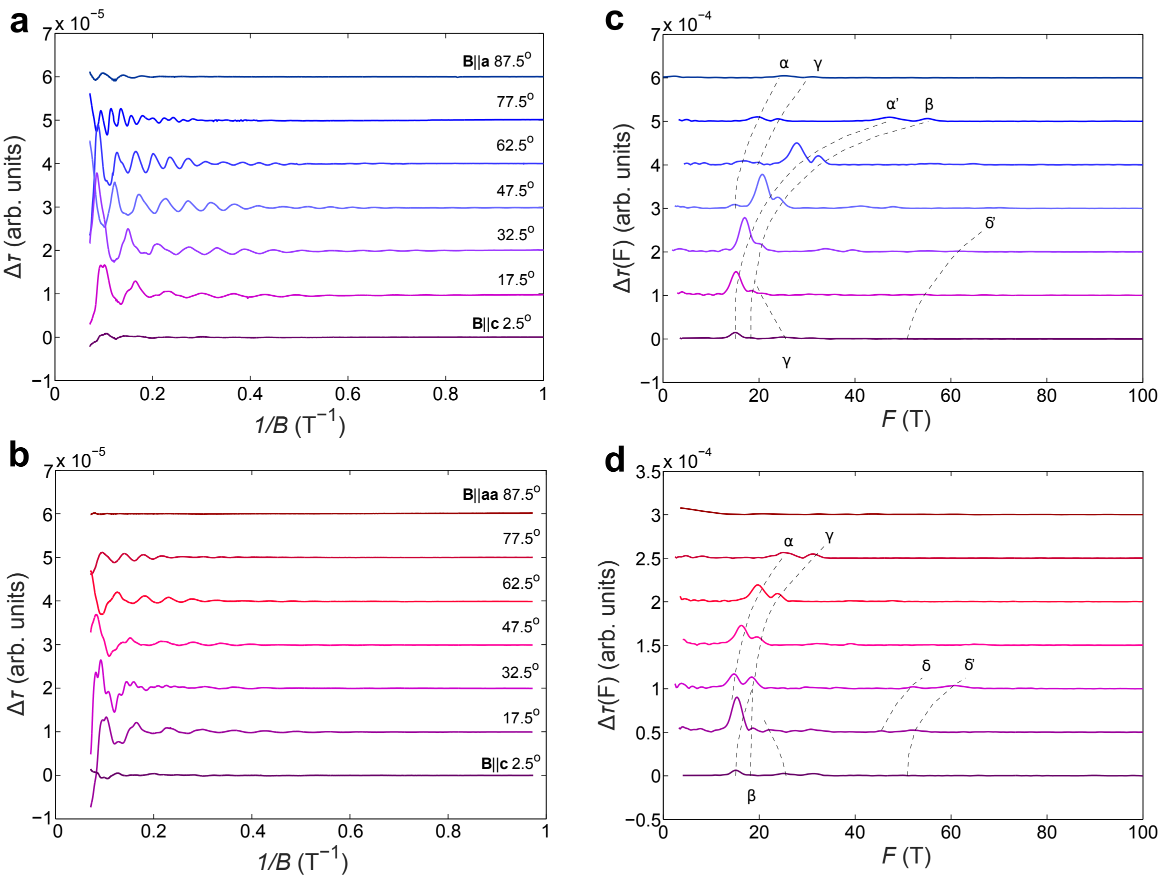

Supplementary Figure 7: Angular dependence of de Haas-van Alphen torque signals. (a) and **(b)** show the magnetic torque de Haas-van Alphen signals at 2 K plotted against the inverse magnetic field for magnetic fields applied in the (100) and (110)-plane respectively. The angles quoted are with respect to the crystallographic **c**-axis. **(c)** and **(d)** show the respective Fourier transforms and characteristic spectra of the de Haas-van Alphen oscillations on the left. Dashed lines are guides to the eye, showing the angular dependence of the individual extremal orbits connected to the E1 and H1 Fermi surface.

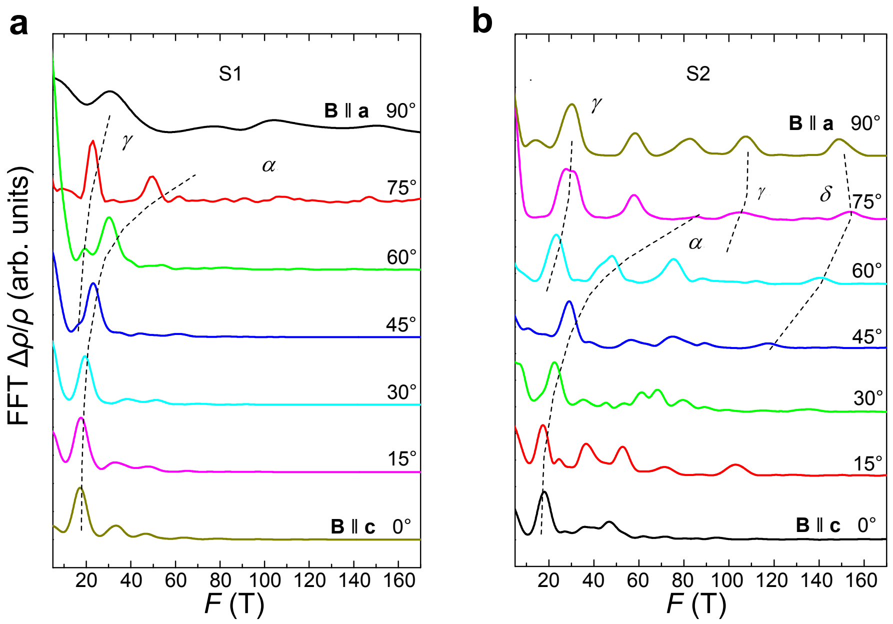

Supplementary Figure 8: Angular Dependence of SdH Signals for S1 and S2. **(a)** shows the Fourier transforms of S1 at different magnetic field angles within the (100)-plane. **(b)** shows the same data for S2.

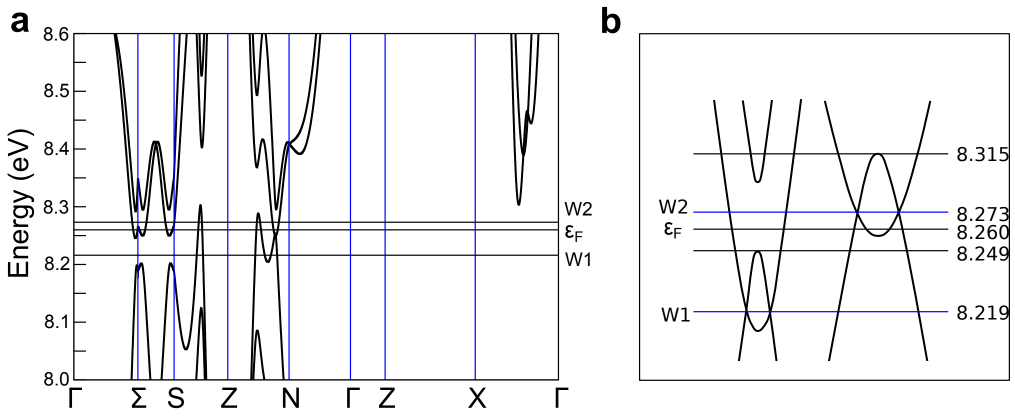

Supplementary Figure 9: ***Ab initio*** **band structure of TaP.** **(a)** shows the *ab initio* band structure along the major directions. Here the horizontal lines correspond to the energies of the W1 and W2 Weyl point as well as the Fermi energy of best fit. **(b)** shows a schematic of the dispersion along the line connecting the individual Weyl point pairs, highlighting the position of the Fermi energy relative to the Weyl points.

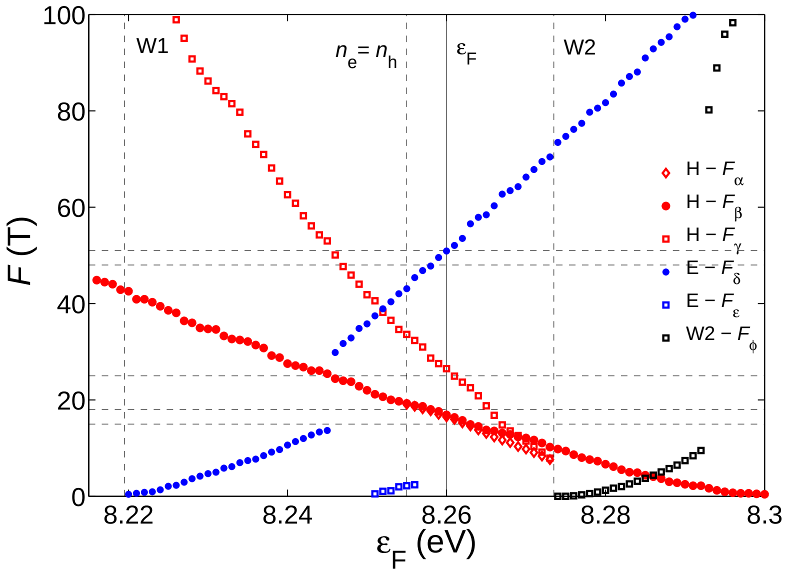

Supplementary Figure 10: Energy dependence of the extremal Fermi surface orbits for B||c. The graph shows the energy dependence of the extremal orbit size. Here the red, blue and black symbols are the respective extremal orbits of the banana shaped hole and electron pocket. The horizontal dashed lines mark the measured dHvA-frequencies for **B**||**c**, whilst the vertical dashed and solid lines represent the Weyl point energies, charge neutral point and energy of the best fitting dHvA frequencies, as shown in Figure 1c of the main text.

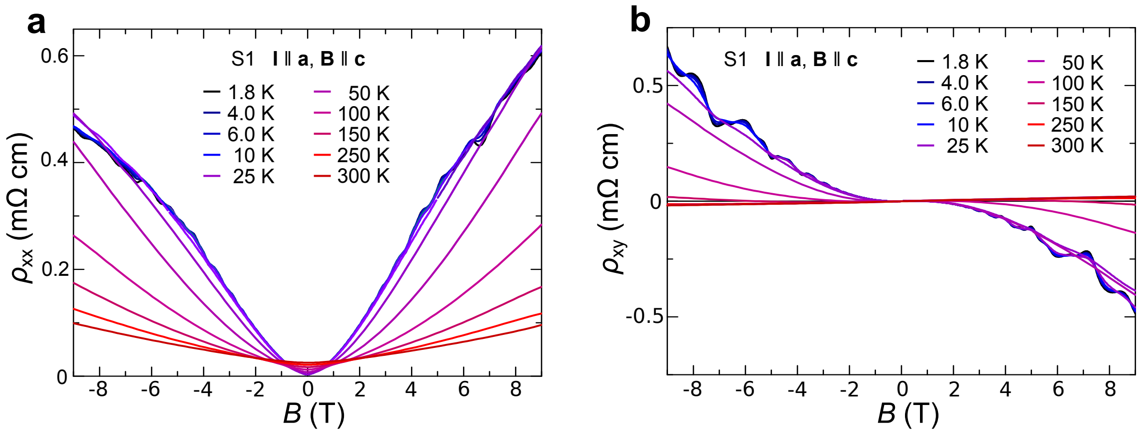

Supplementary Figure 11: Magneto- and Hall resistance. **(a)** shows the unsymmetrized magnetic field dependence of the transverse resistance of sample S1 for $\mathbf{B} \perp \mathbf{I}$ at different temperatures, **(b)** shows the Hall resistivity ρ_{xy} of sample S1 at the same temperatures.

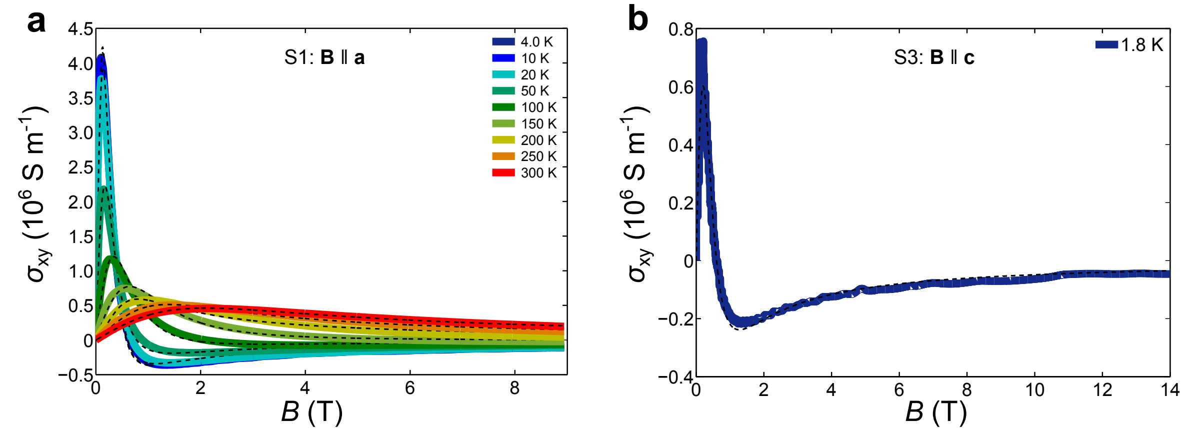

Supplementary Figure 12: Hall conductivity and two-band model fits. **(a)** and **(b)** show the Hall conductivity, σ_{xy} of sample S1 and S3 respectively for $\mathbf{B}||\mathbf{c}$. Solid lines correspond to experimental data whereas dashed lines denote fits to the two-band model.

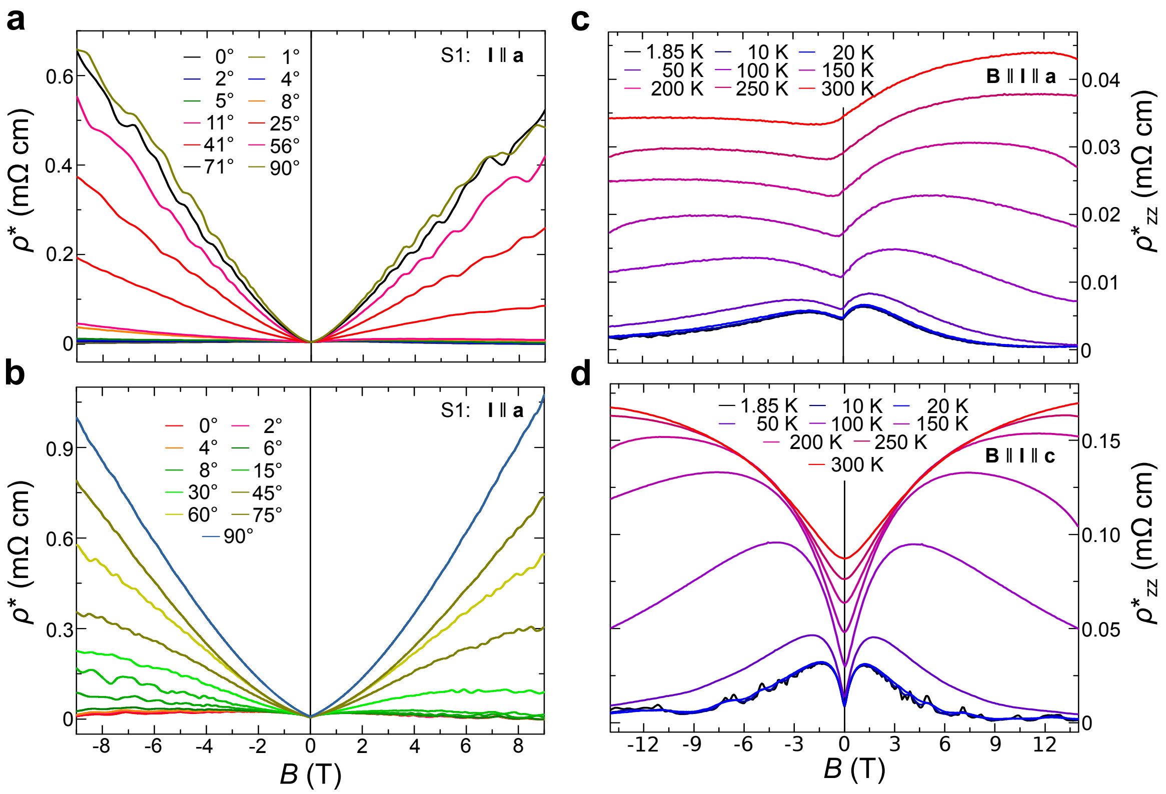

Supplementary Figure 13: Unsymmetrized magnetic field dependences of the resistances. **(a)** and **(b)** show the angular dependence of the magnetoresistance for sample S1 and S2. Here 0° refers to $\mathbf{B}||\mathbf{I}$. **(c)** and **(d)** show the temperature dependences of the negative magnetoresistance of both samples for $\mathbf{B}||\mathbf{I}$.

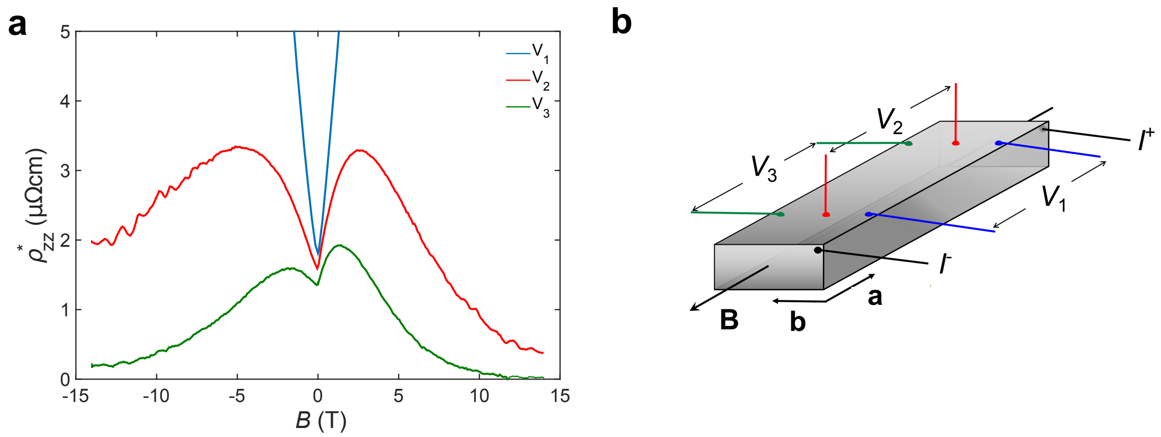

Supplementary Figure 14: Contact dependence of the apparent longitudinal resistivity. **(a)** shows the measured voltage drop of sample S4 for three voltage contact pairs. The geometry of the contacts is shown in **(b)**.

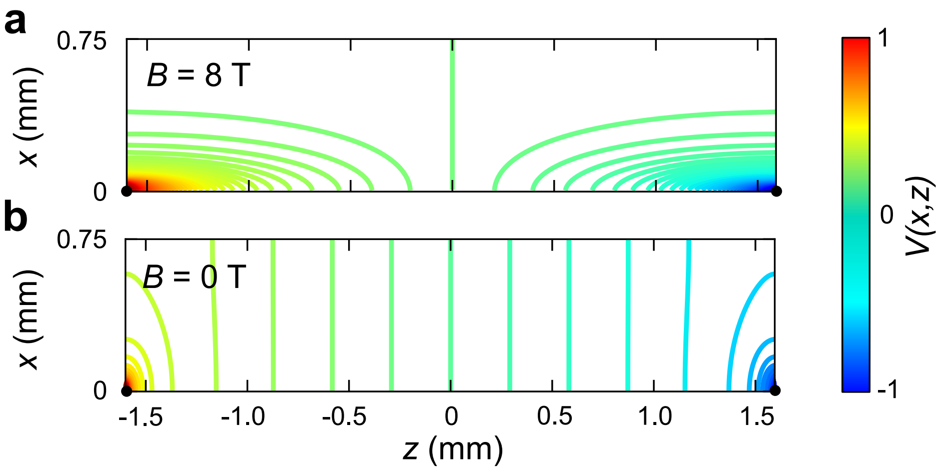

Supplementary Figure 15: Current jetting in TaP. The graph shows the simulated potential distributions of sample S4 for magnetic fields applied parallel to the current contacts at zero field (**a**) and $8\ \mathrm{T}$ (**b**) respectively. The magnetic field drives a redistribution of the current density leading to a focused current jet between the current contacts parallel to the magnetic field and strong bending of the equipotential lines. The black dots show the current source and drain contacts.

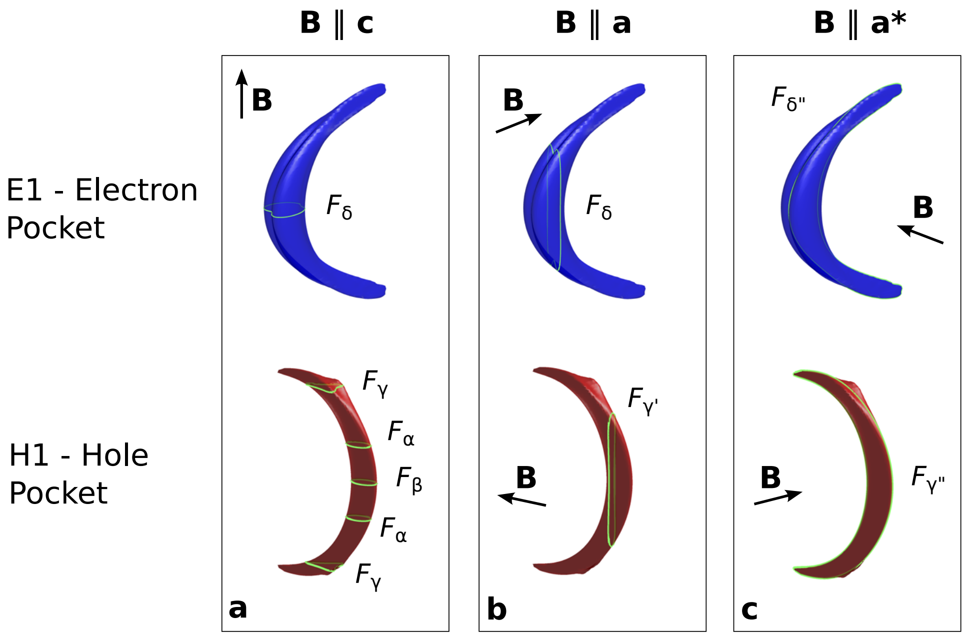

Supplementary Figure 16: Extremal orbits of the E1 and H1 pocket. **(a)** shows the extremal orbits for the magnetic field applied along the **c**-axis. Due to the four-fold rotational symmetry of the tetragonal Brillouin zone, Fermi surface pockets lying in the magnet field tilting plane and perpendicular to it yield the extremal orbits shown in **(b)** and **(c)** when the magnetic field is applied along the crystallographic **a**-axis.

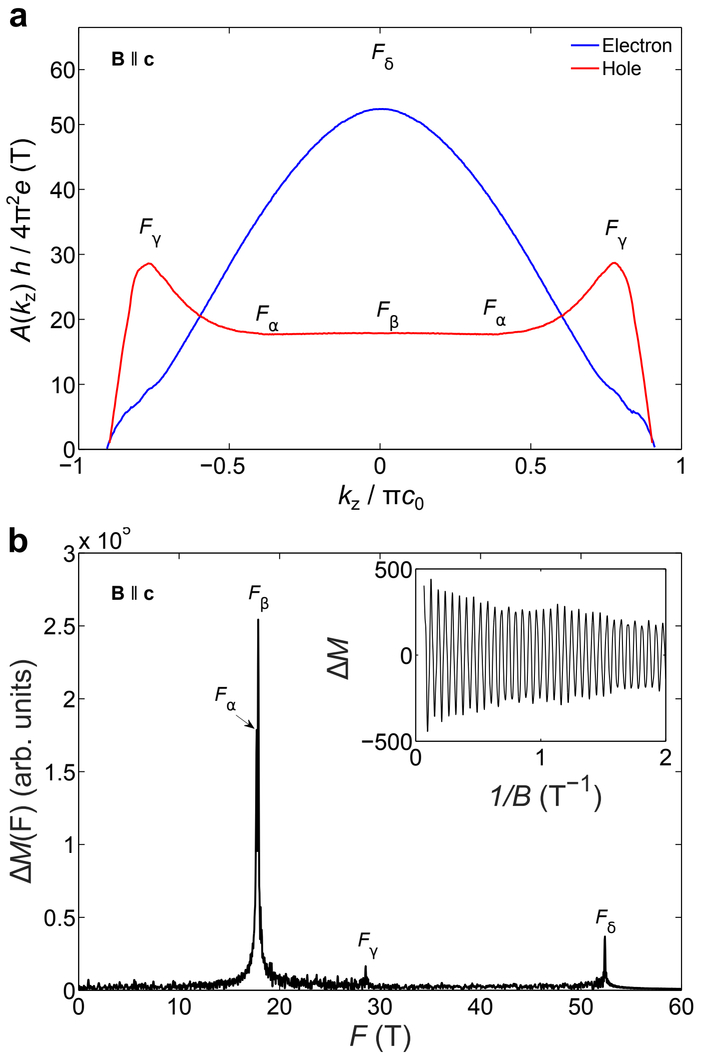

Supplementary Figure 17: Simulated de Haas-van Alphen (dHvA) signal. **(a)** shows the k_z dependence of the Fermi surface cross-section of the electron and hole pocket for $\mathbf{B}||\mathbf{c}$. Here the extremal cross-sections are marked with the name of their respective quantum oscillation frequencies. **(b)** shows the Fourier transform of the dHvA signal of the electron and hole pocket as determined by Supplementary Equation 2. The inset shows the raw dHvA signal.

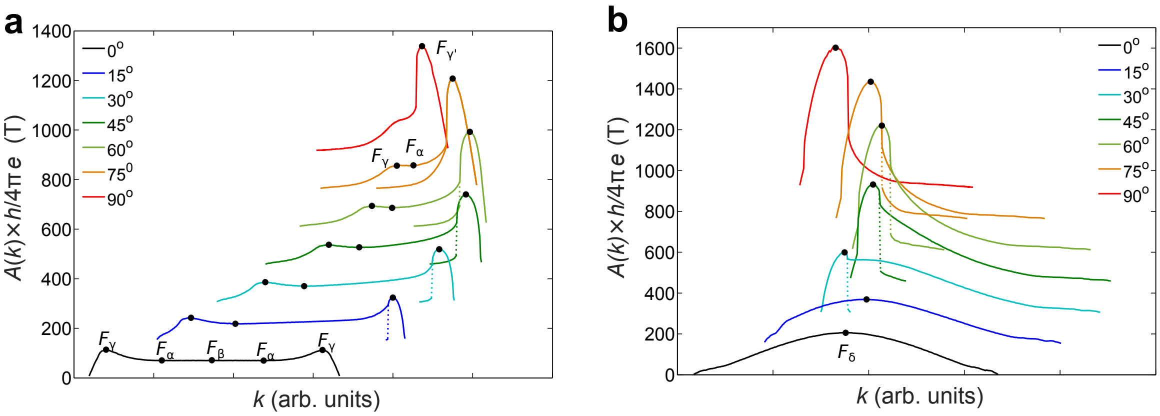

Supplementary Figure 18: Angle-dependent Fermi surface cross sections for B $\in (100)$**.** **(a)** and **(b)** show the $\mathbf{k}||\mathbf{B}$ dependence of the Fermi surface cross section of the hole (H) and electron (E) pocket for different magnetic field directions in the (100)-plane. Magnetic field angles are measured relative to the **c**-axis. Extremal Fermi surface orbits are marked with black dots and their respective names. Curves are shifted for clarity.

Supplementary Tables

Supplementary Table 1: Quantum oscillation parameters as determined from resistivity (Shubnikov-de Haas) and magnetization (de Haas-van Alphen) measurements along the crystallographic **a** and **c** axis. The precise values in the second line are the theoretical frequencies and effective masses. The effective masses are given in units of the free electron mass m_0.

Direction		Hole Pocket					Electron Pocket
		α	β	γ	γ′	γ″	δ
**B**‖**c**	F	(15 ± 1) T<br>17.6 T	(18 ± 1) T<br>17.8 T	(25 ± 1) T<br>28.0 T			(45 ± 2) T<br>(51 ± 2) T<br>52.7 T
	$\frac{m^*}{m_0}$	0.021 ± 0.003<br>0.07	0.05 ± 0.01<br>0.06	0.16			0.11 ± 0.01<br>0.18
**B**‖**a**	F	(26 ± 1) T		(34 ± 2) T	(105 ± 4) T<br>106 T	(295 ± 5) T<br>340 T	(148 ± 5) T<br>174 T
	$\frac{m^*}{m_0}$	0.10 ± 0.03		0.13 ± 0.03	0.35 ± 0.03<br>0.33	1.24	0.4 ± 0.1<br>0.41

Supplementary Notes

Supplementary Note 1

Effective Mass Fitting. By fitting the temperature dependence of the Shubnikov-de Haas (SdH) and de Haas-van Alphen (dHvA) amplitudes to the Lifshitz-Kosevich temperature reduction term[1,2](see Supplementary Fig. 6):

$$\frac{\delta\sigma_{\mathrm{xx}}}{\sigma_{\mathrm{xx}}}(T) \propto \delta\chi(T) \propto \frac{14.69 \times m^{*}T/B}{\sinh(14.69 \times m^{*}T/B)}, \qquad \text{(Supplementary Equation 1)}$$

the effective masses of the strongest dHvA-signals were calculated for magnetic fields applied along both crystallographic axes. The resulting masses are shown in Supplementary Table 1 together with the values predicted by band structure calculations. Temperature dependencies of the raw SdH and dHvA signals can be found in Supplementary Fig. 5 and 6 and Fig. 2b) of the main text.

For magnetic fields applied along the **c** axis, we find electron effective masses of $m^{*}_{\delta} = (0.11 \pm 0.01)\ m_0$ and hole effective masses of $m^{*}_{\alpha} \approx (0.021 \pm 0.003)\ m_0$ and $m^{*}_{\beta} \approx (0.05 \pm 0.01)\ m_0$ for the α and β-orbit respectively, where m_0 is the free electron mass. Both electron and the β-orbit effective masses are connected to extremal belly orbits around the center of their respective Fermi surface pockets in the $k_{\mathrm{z}} = 0$-plane. On tilting the magnetic field away from the **c** axis both effective masses increase with increasing extremal orbit size. We find that both hole effective masses double before we loose their signature 75° out of the **c** axis. The electron effective mass, on the other hand, is enhanced by a factor four along the **a** axis.

The effective masses determined from band structure calculations (see Supplementary Table 1) are of the same order as our experimentally obtained values. The theoretical band masses were directly determined from the slope of the extremal Fermi surface orbits with respect to the Fermi energy (see Supplementary Fig. 10), where $dF/d\epsilon_\mathrm{F} = m^*/e\hbar$.

Supplementary Note 2

Reconstruction of the Fermi Surface Topology. In order to reconstruct the full Fermi surface topology of TaP, the angular dependence of the quantum oscillation frequencies was determined from electrical resistance and magnetic torque measurements at $1.8\ \mathrm{K}$ in magnetic fields up to $14\ \mathrm{T}$. A PPMS rotator option was used to sample the extremal orbits for magnetic fields applied in the (100) and (110)-plane.

dHvA-signals from torque were obtained by background subtracting third order polynomials in the magnetic field range of 0.6 to $14.0\ \mathrm{T}$ (Supplementary Fig. 7a) and b)). Plotted against reciprocal magnetic field the remaining quantum oscillations were Fourier transformed to produce the quantum oscillation spectra (Supplementary Fig. 7c) and d). Fig. 1c) of the main text shows the frequencies determined from the angular dependent SdH and dHvA measurements.

Using *ab initio* band-structure calculations, we were able to identify the Fermi surface topology and Fermi energy matching our measured quantum oscillation frequencies. Supplementary Figure 10 shows the band structure and theoretical energy dependence of the extremal orbits of TaP for $\mathbf{B}||\mathbf{c}$. As can be seen, the energy of best fit lies $5\ \mathrm{meV}$ above the charge neutrality point. Thus TaP

is slightly electron doped as evidenced by the Hall measurements (see Fig. 2c) and d) of the main text).

At a Fermi energy of $\epsilon_{\rm F} = 8.260$ eV the Fermi surface of TaP consists of four banana shaped electron and hole pockets located along the [100]-directions. The β and δ orbit correspond to the quasi-circular belly orbits around the centres of these pockets (see Supplementary Fig. 16 and Fig. 3b) of the main text). For **B**||**c** these orbits lie in the $k_{\rm z} = 0$-plane. On tilting the magnetic field away from the **c**-axis, these orbits tilt along the Fermi surface and become elliptical. The observed $1/cos(\theta)$-like behavior for magnetic fields applied in the (010) and (110)-plane, is an indicator of the almost cylindrical shape near the belly and strong anisotropy of these pockets. Supplementary Figure 17 shows the extremal cross sections and simulated dHvA signal for **B**||**c** based on the Fermi surface topology at 8.26 eV and:

$$\tilde{M} \propto \int_{-\pi/c_0}^{+\pi/c_0} \sin\left(\frac{\hbar}{e}\frac{A(k_{\rm z})}{B}\right) dk_{\rm z}, \qquad \text{(Supplementary Equation 2)}$$

where $A(k_{\rm z})$ are the Fermi-surface cross-sections perpendicular to the magnetic field direction [3]. The α and γ frequencies, on the other hand, originate from extremal orbits of the H1-pocket closer to the W2 Weyl-points (see Supplementary Fig. 16 - 17 and Fig. 3b) of the main text). Here the bumps of the H1 pocket, extending around the W2 Weyl-points, generate a maximal extremal orbit γ running on top of the bumps and a neck orbit α closer to the center of the Fermi surface.

When the magnetic field is applied along the **c**-axis the two external α neck and γ belly orbits, at either end of H1, are degenerate. By tilting the magnetic field in the plane of the Fermi surface pocket, away from the **c** axis, this degeneracy is lifted and the orbits split into larger (prime orbits)

and smaller orbits (see Supplementary Fig. 18). β being a belly orbit does not split. At a small critical angle of about 3° the larger α' neck and central β orbit merge and vanish. This is the analog of the first Yamaji angle in a quasi two-dimensional electron system [4]. On tilting the magnetic field even further, the splitting of the external γ and γ'-orbits increases before the smaller γ orbit merges with the remaining α neck orbit. Beyond 80°, the Fermi surface shows only one extremal orbit γ'.

Due to the fourfold symmetry of the tetragonal Brillouin zone, the hole pockets which are located at 90° to the plane in which the magnetic field is tilted give rise to three more frequency branches (double primed, dashed lines in Fig. 1c) of the main article). All of these orbits follow a quasi-two dimensional $1/cos(\theta)$ behaviour up to large angles. On increasing the polar field angle, the β" orbit vanishes as the two α" orbits are pushed towards the centre of the Fermi surface where they merge. In close proximity to the **a** axis the remaining α" orbit vanishes, as the two γ"-orbits merge. The new γ" orbit encloses the entire H1 Fermi surface (see Supplementary Fig. 16). The E1 electron pockets on the other hand, give rise to an ellipsoid like angular dependence of the extremal orbit size. Minor differences are only visible at large angles where the banana-shape leads to a flatter angular dependence around the **a** axis.

Compared to the band structure calculation we observe a more pronounced F_α neck-orbit. This leads to a larger splitting of F_α and F_β and increases the critical angle for the α and γ orbit. Both frequencies can observed up to $\mathbf{B}||\mathbf{a}$. The splitting of F_α and F_β, i.e. their frequency difference varies slightly between different band-structure calculation methods and is beyond the resolution of our band structure calculations. Similarly the splitting observed in δ is not reproduced by the

calculation. Here fine structures like a central waist in the electron pocket might give rise to the observed splitting.

Supplementary Note 3

Scattering Times and Mobilities. The scattering time τ, mean free path l and charge carrier mobility μ can be determined from the magnetic field dependence of the quantum oscillation amplitude i.e. the Dingle reduction term [2,12]:

$$\tilde{M}(B) \propto B^{-1/2} \exp\left(-\frac{14.69 \times m^* T_{\mathrm{D}}}{B}\right). \qquad \text{(Supplementary Equation 3)}$$

Here $T_{\mathrm{D}} = \hbar/2\pi k_{\mathrm{B}}\tau$ is the Dingle temperature, which is inversely proportional to the scattering times. In reciprocal field and corrected for the $\sqrt{B}$-prefactor, the Dingle term represents an exponentially decaying envelope function, which results in a Lorentzian line shape observed in the quantum oscillation spectra. As the full-width half-maximum of these Lorentzians lines $\delta F_{1/2}$ is directly related to the Dingle temperature by $T_{\mathrm{D}} = \delta F_{1/2}/(14.694\pi m^*)$, the line width can be used to determine the Dingle temperature and scattering times in systems with complex dHvA-spectra.

The Dingle temperature of the hole pocket has been extracted from the Lorentzian line width. By varying the magnetic field window, over which the data is Fourier transformed, at low fields, we confirmed that the observed linewidths show no significant sinc-function contribution originating from a finite window size. Here, the lowest magnetic field was chosen such that the quantum oscillation amplitude has decayed to beneath the noise level. A double Lorentzian fit was applied to the F_{α} -F_{β} double peak to extract the linewidth of both signals. Simulations showed that for the

given window size and decay constants the sinc-contribution accounts for 1 and 21 $\%$ of the total linewidths of F_α and F_β respectively. Based on the intrinsic linewidths, Dingle temperatures of 1.5 ± 0.3 K and scattering times of $(8 \pm 2) \times 10^{-13}$ s were calculated for both orbits. Using the fitted effective masses we can calculate the charge carrier mean free path and mobility $\mu = \tau e/m^*$ of the hole charge carriers. We find that the hole mobility is $\mu_\mathrm{h} \approx (3.2 \pm 0.5) \times 10^4\ \mathrm{cm^2V^{-1}s^{-1}}$. Taking into account the measured dHvA-frequencies, effective masses and assuming a parabolic band, the mean free path of the holes can be calculated by applying: $l = v_\mathrm{F} \times \tau$ with $v_\mathrm{F} = \sqrt{\frac{2e\hbar}{m^{*2}}F}$. Here, we find a mean free path of $l_\mathrm{h} = 0.4\,\mu\mathrm{m}$. Performing the same analysis on the electron pocket was not possible due to its comparably small signal size.

Supplementary Note 4

Hall Effect and Charge Carrier Density and Mobility. Supplementray Figure 11 shows the tranverse and Hall resistivity, ρ_xy, of the samples S1. The Hall coefficients of both TaP samples are initially positive and change sign above 1 T. The Hall sign reversal field is strongly temperature dependent. This nonlinear magnetic field dependence shows the presence of two competing charge carrier types in TaP and can be used to obtain the respective charge carrier densities. In the case where **B**$||$**c**, the transverse and Hall conductivities are related to the resistivities via [13]:

$$\sigma_\mathrm{xx} = \frac{\rho_\mathrm{xx}}{\rho_\mathrm{xy}^2 + \rho_\mathrm{xx}^2} \qquad \text{and} \qquad \sigma_\mathrm{xy} = -\frac{\rho_\mathrm{xy}}{\rho_\mathrm{xy}^2 + \rho_\mathrm{xx}^2} \qquad \text{(Supplementary Equation 4)}$$

The Hall conductivities are fitted by a two-band model using:

$$\sigma_\mathrm{xy} = \left[n_\mathrm{h}\mu_\mathrm{h}^2 \frac{1}{1 + (\mu_\mathrm{h}B)^2} - n_\mathrm{e}\mu_\mathrm{e}^2 \frac{1}{1 + (\mu_\mathrm{e}B)^2} \right] eB \qquad \text{(Supplementary Equation 5)}$$

$$\sigma_{\mathrm{xx}}(B=0) = [n_{\mathrm{h}}\mu_{\mathrm{h}} + n_{\mathrm{e}}\mu_{\mathrm{e}}]e, \qquad \text{(Supplementary Equation 6)}$$

where n_{h} and n_{e} are the hole and electron densities, μ_{h} and μ_{e} are the hole and electron mobilities respectively, and e is the elementary charge.

Supplementary Figure 12a) and b) show the two-band model fits of the Hall conductivities of samples S1 and S3. Their Hall conductivity is well described by the two-band model over the entire temperature and magnetic field range. Slight deviations of the fits from the data originate from the uncertainty of the contact geometry influencing the absolute values of ρ_{xx} and ρ_{xy}. The resulting charge carrier densities and mobilities are shown in Fig. 2 of the main text. Here, the carrier densities remain almost constant within the error bars of our measurements. However, we observe signs of a cross over from electron to hole dominated transport at 150 K. This is also reflected in the shift of the Hall conductivity zero towards higher fields and beyond the field range studied in this article.

Supplementary Note 5

Angular Dependence of the Magnetoresistance. Owing to the large charge carrier mobility of TaP (see also Fig. 2d) of the main text), we observe a high magnetoresistance (MR) for $\mathbf{B} \perp \mathbf{I}$. As an example Supplementary Fig. 11 shows the transverse resistivity of samples S1 for $\mathbf{B}\perp\mathbf{I}$ at selected temperatures between 2 and 300 K and magnetic fields up to 9 T. Up to 25 K, we observe an almost constant strong positive $\mathrm{MR} = (\rho_{\mathrm{xx}}(B) - \rho_0)/\rho_0 = 1.8 \times 10^4$ % at 9 T. Above 25 K, MR is dropping to about 100 % at 9 T and room temperature due to the decreasing charge carrier

mobility (see Supplementary Note 5).

The longitudinal resistivities, ρ_{zz}, were measured from room temperature down to 1.8 K for magnetic fields from -14 to 14 T. Supplementary Figure 13 shows the temperature and angular dependence of the longitudinal resistivity of samples S1 and S2. Here $\rho^{*}_{\mathrm{zz}} = V_{\mathrm{z}}/I_{\mathrm{z}} \times A/l$ is the apparent longitudinal resistivity for an assumed homogenous current distribution in the sample.

For perfect alignment of magnetic field and current to better than 2°, a negative MR* can be observed. Its magnitude depends strongly on the precise location of the voltage contacts with respect to the current contacts.

Supplementary Figure 14 shows the longitudinal resistivity of sample S4 measured by three different voltage contact pairs. In zero field, all three contact pairs show approximately the same resistivity, indicating a homogenous current flow through the sample. On increasing the magnetic field, the measured resistivities diverge. It can be seen that the negative MR is largest for the contact pairs furthest away from the current contacts, whereas the contacts which are in-line with the current contacts show a strongly positive MR. Such a behavior can be explained by a magnetic field induced current redistribution in the sample, where the current flows homogeneously through the sample in zero field and concentrates on a narrow path directly between the current contacts in high fields (see also Supplementary Fig. 15). This phenomenon is called current jetting, and was first observed in chromium and tungsten [14]. It arises due to a strong anisotropy of the longitudinal and transverse conductivity [14–16]. Under a large magnetic field, the longitudinal conductivity $\sigma_{\mathrm{zz}} \gg \sigma_{\mathrm{xx}}$ of a high mobility metal becomes much larger than its transverse conductivity. A cur-

rent injected through a single point into the sample will thus primarily follow the magnetic field and form a current jet. Its width depends to first approximation on the charge carrier mobility as: $\theta \approx 2\tan^{-1}\left(\sqrt{3}/\omega_c\tau\right)$ leading to an opening angle of the current jet of approximately 3° at 14 T in TaP [17]. On applying a magnetic field the current is drawn from the outer parts of the sample and focused between the current contacts. Thus the voltage measured at the contact pair furthest away from the current contacts drops due to the vanishing current in this region of the sample, whilst the voltage increases between the current contacts. However neither of these contacts measure the correct intrinsic longitudinal resistivity.

Supplementary Note 6

Theoretical Procedure of the Current Jetting Calculations We now outline the procedure to generate the theoretical curves shown in Supplementary Fig. 15 and Fig. 4d) of the main text. For the sample with the geometry shown in Supplementary Fig. 14 we first solve the Laplace equation following [18], taking into account the position and size of the contacts. The resulting potential distribution depends on the transverse and longitudinal conductivity as well as the sample geometry. In this calculation we assume that the effect of the Hall effect contribution is small and can be neglected i.e. $\rho_{\mathrm{xy}} = 0$. The plotted curves for different contact configurations are shown in Fig. 4d) of the main text. The corresponding potential distributions at the sample surface for magnetic fields of 0 T and 8.02 T are shown in Supplementary Fig. 15. In order to obtain these plots we have i) used the experimentally measured transverse conductivity as an input ii) assumed a constant longitudinal conductivity fixed to match the zero magnetic field value measured with

the V_2 arrangement and iii) taken the experimentally measured geometry of the sample which is $3.2 \times 0.75 \times 0.57\ \mathrm{mm}^3$ and contact size of $25 \times 25\ \mu\mathrm{m}^2$. We note that by applying i), ii) and iii) there are no free parameters. It is therefore remarkable how the qualitative features that are seen in experiments, i.e. positive and negative magnetoresistance depending on the contact position is captured by our modeling.

Supplementary References

1. Lifshitz, I. & Kosevich, A. Theory of Magnetic Susceptibility in Metals at Low Temperature. *Sov. Phys. JETP* **2**, 636–645 (1956).

2. Shoenberg, D. *Magnetic Oscillations in Metals* (Cambridge University Press, 2009).

3. Bergemann, C., Julian, S. R., Mackenzie, A. P., NishiZaki, S. & Maeno, Y. Detailed Topography of the Fermi Surface of Sr_2RuO_4. *Phys. Rev. Lett.* **84**, 2662–2665 (2000).

4. Yamaji, K. On the Angle Dependence of the Magnetoresistance in Quasi-Two-Dimensional Organic Superconductors. *J. Phys. Soc. Jpn.* **58**, 1520–1523 (1989).

5. He, L. *et al.* Quantum transport evidence for the three-dimensional Dirac semimetal phase in Cd_3As_2. *Rhys. Rev. Lett.* **113**, 246402 (2014).

6. Weng, H., Fang, C., Fang, Z., Bernevig, B. & Dai, X. Weyl Semimetal Phase in Noncentrosymmetric Transition-Metal Monophosphides. *Phys. Rev. X* **5**, 011029 (2015).

7. Hubbard, S., Kershaw, T., Usher, A., Savchenko, A. & Shytov, A. Millikelvin de Haas van Alphen and Magnetotransport Studies of Graphite. *Phys. Rev. B* **83**, 035122 (2011).

8. Onsager, L. Interpretation of the de Haas-van Alphen effect. *Phil. Mag.* **43**, 1006–1008 (1952).

9. Wang, Z. *et al.* Helicity protected ultrahigh mobility Weyl fermions in NbP. Preprint at `http://arxiv.org/abs/1506.00924` (2015).

10. Luo, Y. *et al.* A novel electron-hole compensation effect in NbAs. Preprint at http://arxiv.org/abs/1506.01751 (2015).

11. Yang, X., Li, Y., Wang, Z., Zhen, Y. & Xu, Z. Observation of Negative Magnetoresistance and nontrivial π Berry phase in 3D Weyl semi-metal NbAs. Preprint at http://arxiv.org/abs/1506.02283 (2015).

12. Dingle, R. Some Magnetic Properties of Metals. II. The Influence of Collisions on the Magnetic Behaviour of Large Systems. *Proc. Roy. Soc. A* **211**, 517–525 (1952).

13. Singleton, J. *Band Theory and Electronic Properties of Solids* (Oxford Master Series in Physics, 2001).

14. Reed, W., Blount, E., Marcus, J. & Arko, A. Anomalous Longitudinal Magnetoresistance in Metals. *J. Appl. Phys.* **42**, 5453 (1971).

15. Yoshida, K. A Geometrical Transport Model for Inhomogenous Current Distribution in Semimetals under High Magnetic Field. *J. Phys. Soc. Jpn.* **40**, 1027–1034 (1976).

16. Ueda, Y. & Kino, T. Anisotropy of the Apparent Resistivity in High-Purity Aluminium Single Crystals in Longitudinal Magnetic Field. *J. Phys. Soc. Jpn.* **48**, 1601–1606 (1980).

17. Pippard, A. *Magnetoresistance in Metals* (Cambridge University Press, 1989).

18. Yoshida, K. Transport of spacially inhomogeneous current in a compensated metal under magnetic fields. III. A case of bismuth in longitudinal and transverse magnetic fields. *J. Appl. Phys.* **51**, 4226–4233 (1980).
